# Metagenomic Analysis of Serum Microbe-Derived Extracellular Vesicles and Diagnostic Models to Differentiate Ovarian Cancer and Benign Ovarian Tumor

**DOI:** 10.3390/cancers12051309

**Published:** 2020-05-21

**Authors:** Se Ik Kim, Nayeon Kang, Sangseob Leem, Jinho Yang, HyunA Jo, Maria Lee, Hee Seung Kim, Danny N. Dhanasekaran, Yoon-Keun Kim, Taesung Park, Yong Sang Song

**Affiliations:** 1 Department of Obstetrics and Gynecology, Seoul National University College of Medicine, Seoul 03080, Korea; seikky@naver.com (S.I.K.); marialeemd@gmail.com (M.L.); bboddi0311@gmail.com (H.S.K.); 2 Department of Statistics, Seoul National University, Seoul 08826, Korea; thisisnykang@gmail.com; 3 Department of Core Technology, R&D Center, LG Household & Healthcare, Seoul 07795, Korea; leemss@ajou.ac.kr; 4 MD Healthcare Inc., Seoul 03923, Korea; jhyang@mdhc.kr (J.Y.); ykkim@mdhc.kr (Y.-K.K.); 5 Cancer Research Institute, Seoul National University College of Medicine, Seoul 03080, Korea; whgusdk25@snu.ac.kr; 6 Stephenson Cancer Center, University of Oklahoma Health Sciences Center, Oklahoma City, OK 73104, USA; Danny-Dhanasekaran@ouhsc.edu

**Keywords:** ovarian neoplasms, ovarian carcinoma, extracellular vesicle, microbiome, metagenomic analysis, diagnostic model

## Abstract

We aimed to develop a diagnostic model identifying ovarian cancer (OC) from benign ovarian tumors using metagenomic data from serum microbe-derived extracellular vesicles (EVs). We obtained serum samples from 166 patients with pathologically confirmed OC and 76 patients with benign ovarian tumors. For model construction and validation, samples were randomly divided into training and test sets in the ratio 2:1. Isolation of microbial EVs from serum samples of the patients and 16S rDNA amplicon sequencing were carried out. Metagenomic and clinicopathologic data-based OC diagnostic models were constructed in the training set and then validated in the test set. There were significant differences in the metagenomic profiles between the OC and benign ovarian tumor groups; specifically, genus *Acinetobacter* was significantly more abundant in the OC group. More importantly, *Acinetobacter* was the only common genus identified by seven different statistical analysis methods. Among the various metagenomic and clinicopathologic data-based OC diagnostic models, the model consisting of age, serum CA-125 levels, and relative abundance of *Acinetobacter* showed the best diagnostic performance with the area under the receiver operating characteristic curve of 0.898 and 0.846 in the training and test sets, respectively. Thus, our findings establish a metagenomic analysis of serum microbe-derived EVs as a potential tool for the diagnosis of OC.

## 1. Introduction

Ovarian cancer (OC) is the deadliest gynecologic cancer worldwide [1]. In the United States, the number of new cases of OC and cancer deaths from OC in 2019 were estimated to be 22,530 (2.5% of all female cancers) and 13,980 (4.9% of female cancer deaths), respectively [2]. In Korea, the incidence of OC has been increasing gradually [3]. Owing to a lack of specific symptoms and effective screening tools, the majority of OC cases are diagnosed at an advanced stage, resulting in a high recurrence and mortality rate [4]. Among the various histologic types of OC, the majority (90%) are epithelial OC.

For women with adnexal masses, distinguishing OC from benign ovarian tumors is an important issue, as it determines the treatment plan, including the surgical approach. The detection tools currently available for OC are serum cancer antigen 125 (CA-125) levels, ultrasonography, computed tomography (CT) scans, and magnetic resonance imaging (MRI). Combinations of modalities provide better diagnostic performance for identifying OC than each modality alone [5]. The risk of malignancy index (RMI) scoring system, consisting of serum CA-125, menopausal status, and ultrasound features, as well as the risk of ovarian malignancy algorithm (ROMA), a biomarker-based algorithm consisting of serum CA-125 and human epididymis protein 4 (HE4), have been developed [6,7]. Both RMI and ROMA are reliable tools and perform equally well in differentiating OC from adnexal masses [8,9]. However, considering that their diagnostic performance and accuracy differ among the prospective cohort studies, further improvements in differentiating adnexal masses are still needed [10,11,12].

Microbiota, a microbial environmental factor that we are constantly exposed to, has emerged as a link between the host and various cancer types. Human microbiome studies have revealed that significant differences in microbiota composition are associated with oral, esophageal, pancreatic, and colorectal cancers [13,14,15,16]. Although the exact underlying mechanisms are still not well-understood, microbe-induced inflammation is thought to trigger changes in the tumor microenvironment, promoting tumorigenesis [17,18]. Advances in the sequencing technique of microbial genomes have expanded microbiome data and extended our understanding on microbiota-host interactions. Especially, 20–200 nanometer-sized extracellular vesicles (EVs), constitutively secreted by microbes and detectable in body fluids, are considered to play an important role in such interactions [19,20].

A recent study has compared the microbiome signature between fresh OC tissues (*n* = 25) and normal fallopian tube fimbria tissues (*n* = 25) and suggested that changes in microbial composition might be related to the process of OC development [21]. However, the relationship between serum microbial EVs and OC has yet to be investigated. The relative abundance of certain microbial EVs released in the blood might differ between benign and malignant ovarian tumors, and those differences could be utilized in the differential diagnosis of adnexal masses. Thus, this study aimed at developing diagnostic models to differentiate between OC and benign ovarian tumors through the metagenomic analysis of serum microbial EVs.

## 2. Results

### 2.1. Characteristics of the Study Population

The overall study design is displayed in Figure S1. Table 1 presents the clinicopathologic characteristics of all patients. Although patients in the OC group were significantly older than those in the benign ovarian tumor group (mean, 53.6 vs. 49.4 years; *p* = 0.041), other characteristics such as body mass index (BMI), menopausal status, and comorbidities were similar. After 2:1 random distribution of the patients into training and test sets, OC patients were still older than those with benign ovarian tumors in the training set, whereas patients’ ages were similar in the test set. Both in the training and test sets, no differences in BMI, menopausal status, and comorbidities were observed between the OC and benign ovarian tumor groups.

In the training set, serum CA-125 levels were significantly higher in patients with OC (median, 331.1 vs. 22.3 IU/mL; *p* < 0.001). Among the 110 patients with OC, 39 (35.5%) and 71 (64.5%) were diagnosed with the International Federation of Gynecology and Obstetrics (FIGO) stage I-II and III-IV, respectively. The most common histologic type was high-grade serous carcinoma, which was observed in 54.5% of OC patients. Among the 51 patients with benign ovarian tumor, mucinous cystadenoma (47.1%) was the most common pathologic diagnosis, followed by serous cystadenoma (15.7%).

In the test set, the OC group also showed significantly higher serum CA-125 levels compared to the benign ovarian tumor group (median, 432.3 vs. 20.6 IU/mL; *p* < 0.001). FIGO stage I-II disease was observed in 41.1% of OC patients. The most common histologic types in the OC and benign ovarian tumor groups were high-grade serous carcinoma (50.0%) and serous cystadenoma (28.0%), respectively.

### 2.2. Comparison of Metagenomic Profiles between the Two Groups

**Figure 1:**
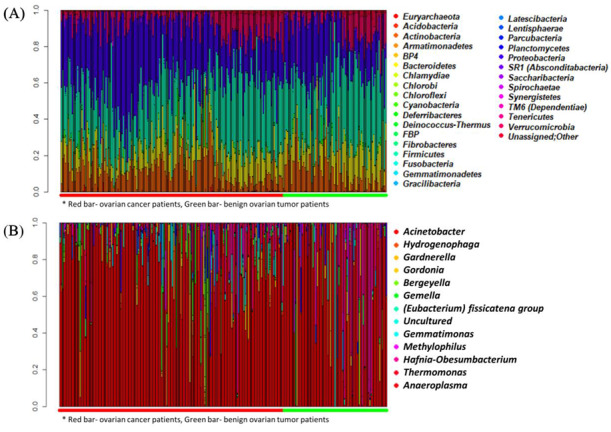
Landscape of metagenomic profiles in all patients. (**A**) Phylum-level composition. (**B**) Genus-level composition. Below the plot, red and green horizontal bars indicate ovarian cancer patients and benign ovarian tumor patients, respectively.

Figure 1 depicts the landscape of the metagenomic profiles of all patients. Figure 1A shows all 31 phyla detected in OC and benign ovarian tumor groups. In the genus-level composition, a total of 587 genera were detected in all patients. Among them, 110 significantly differentially distributed genera identified by at least two statistical methods are displayed with their relative abundance in Figure 1B. Herein, genus *Acinetobacter* showed high relative abundances both in OC and benign ovarian tumor groups. In the training set, 107 of 110 ovarian cancer patients (97.3%) had *Acinetobacter*, while 50 of 51 benign ovarian tumor patients (98.0%) had *Acinetobacter*. In the test set, *Acinetobacter* was found in 98.2% (55/56) and 100.0% (25/25) of OC and benign ovarian tumor groups, respectively.

In general, “genus” is regarded as the lowest level of taxonomy, where unassigned or unclassified microbiome are relatively small. Most previous studies on microbiome analyses have reported metagenomic profiles up to the genus level. Therefore, we investigated further metagenomic profiles of the two groups in the genus level.

In metagenomics, α-diversity and β-diversity are used to overview the distribution of the data composition: α-diversity refers to the richness, evenness, and dominance of taxa in a particular community, while β-diversity means taxonomic differences between the communities [22]. Comparing the genus-level α-diversity, the Shannon index was not different between the OC and benign ovarian tumor groups in the training set (median, 3.294 vs. 3.263; *p* = 0.270), as well as in the test set (median, 3.210 vs. 3.238; *p* = 0.810) (Figure S2). In order to compare β-diversity, we analyzed clustering at the genus level using multidimensional plots. However, these plots did not show distinguished clustering between the OC and benign ovarian tumor groups in the training and test sets (Figure S3).

### 2.3. Development of Diagnostic Models for Ovarian Cancer in the Training Set

Through the metagenomic analyses using various statistical methods, we identified genus-level microbiome biomarkers that were differentially distributed between the OC and benign ovarian tumor groups with statistical significance: Wilcoxon test, Metastats, EdgeR, DESeq2, zero-inflated Gaussian mixture model (ZIG), zero-inflated beta regression (ZIBSeq), analysis of composition of microbiomes (ANCOM), and centered log-ratio transformation and permutation logistic regression model (CLR Perm) identified 1, 98, 3, 8, 447, 56, 1, and 2 biomarkers, respectively, at adjusted *q* values using a false discovery rate (≤ 0.05). Table 2 shows the top 10 microbiome biomarkers identified by each statistical method, in order of overlap.

**Figure 2:**
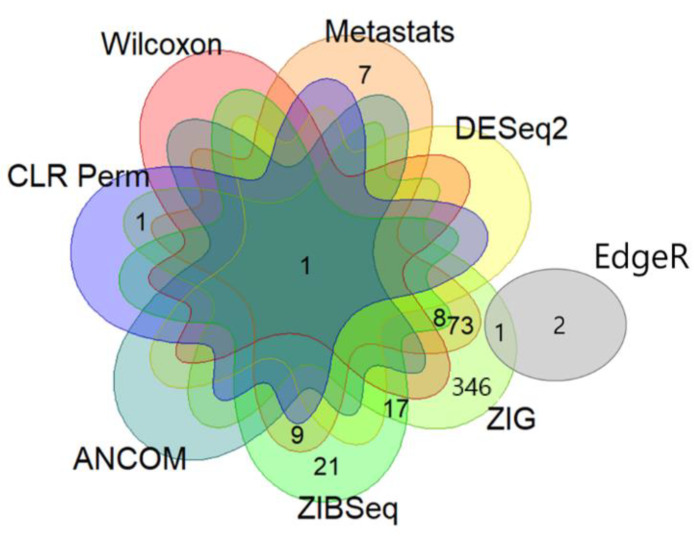
Selection of genus-level microbiome biomarkers. Venn diagram depicts the overlapping of biomarkers among the eight statistical methods.

Next, we examined the overlap of these genus-level microbiome biomarkers among the eight statistical methods (Figure 2). In total, 486 biomarkers were identified to be significantly differentially distributed by at least one statistical method. Among them, 110 and nine markers overlapped at least two and three statistical methods, respectively. *Acinetobacter* was the only common genus identified by seven different statistical analysis methods. Specifically, *Acinetobacter* was significantly more abundant in the OC group than in the benign ovarian tumor group (median (interquartile range), 0.084 (0.037–0.222) vs. 0.033 (0.008–0.075); Wilcoxon rank sum test, *p* < 0.001). Therefore, we selected *Acinetobacter* as the most potential and highly plausible genus-level microbiome biomarker for differentiating OC from benign ovarian tumors.

Combining the relative abundance of *Acinetobacter* with patients’ clinicopathologic variables, we constructed several diagnostic models to differentiate OC from benign ovarian tumors (Table 3). The model composed of age, serum CA-125 levels, and relative abundance of *Acinetobacter* showed 86.4% sensitivity and 78.4% specificity. This model showed a superior area under the receiver operating characteristic curve (AUC; 0.898) than any other models, with less than three of the following variables: age, serum CA-125 levels, and *Acinetobacter*.

### 2.4. Validation of Diagnostic Models for Ovarian Cancer

**Figure 3:**
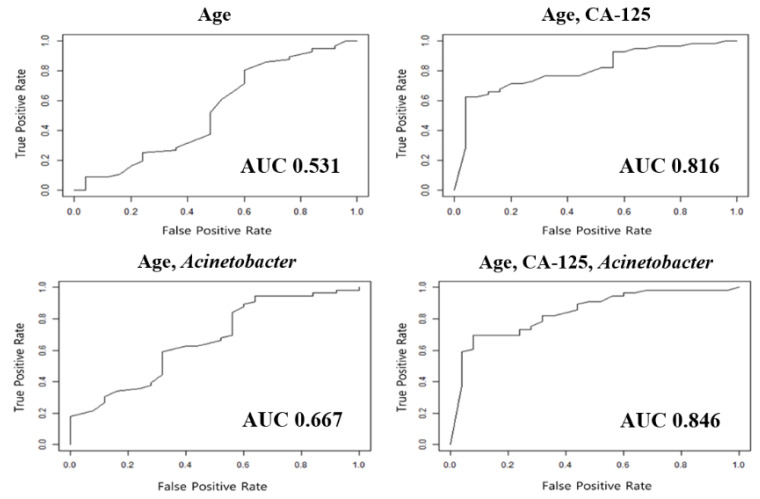
Comparisons of performances among diagnostic models differentiating ovarian cancer from benign ovarian tumors.

The developed diagnostic models were validated in the test set. Among the various models, the model consisting of patients’ ages at diagnosis, initial serum CA-125 levels, and relative abundance of *Acinetobacter* yielded the best diagnostic performance for differentiating OC from benign ovarian tumors as follows: sensitivity, 82.1%; specificity, 68.0%; and AUC, 0.846 (Table 3 and Figure 3).

## 3. Discussion

In the present study, we successfully extracted microbe-derived EVs from the serum samples and characterized the metagenomic profiles of 242 patients: 166 with OC and 76 with benign ovarian tumors. Incorporating the relative abundance of specific microbiomes at the genus level with patients’ ages and serum CA-125 levels, we developed a new diagnostic model to differentiate OC from benign ovarian tumors; this model even showed a better diagnostic performance than those without a microbiome biomarker.

Recently, metagenomic analysis has been noticed as a new approach; it has opened new horizons in the diagnosis of human disease. The Human Microbiome Project, funded by the National Institutes of Health, triggered the broadening of our insights into the microbiome. The relative abundance of certain microbes varies in chronic diseases, such as diabetes, obesity, cardiovascular disease, inflammatory bowel disease, and chronic allergies [23,24]. In various malignancies, disruption in the stability of microbiota or structural microbiome shifts have been reported [13,14,15,16]. However, to date, few studies have examined microbiomes in OC [21].

The current study provides new scientific evidence regarding different distributions of microbiomes in serum EVs between OC and benign ovarian tumors. Unlike previous researchers who used samples obtained from female reproductive organs [21,25], we used patients’ serum samples. Compared to the former, obtaining serum samples is much easier and less invasive; organ harvesting is not required. Considering the fact that an exact diagnosis is confirmed through surgery, a preoperative diagnostic model using serum samples certainly has merit. Therefore, our study shows the potential of serum microbial EVs as a liquid biopsy for the diagnosis of OC.

Interestingly, we found that the genus *Acinetobacter* was significantly more abundant in the OC group than in the benign ovarian tumor group. Moreover, *Acinetobacter* was the only commonly found genus through almost all available statistical analysis methods developed so far. In general, *Acinetobacter baumannii* (*A. baumannii*), a species of the genus *Acinetobacter*, is a pathogen related to human infections, such as pneumonia, blood stream infection, urinary tract infection, and meningitis [26]. Infection with *Acinetobacter* is also common in cancer patients, and a relationship between *A. baumannii* and poor survival outcomes was also reported among patients with various cancer types [27,28]. Similar to our study, Zhou et al. showed that *Acinetobacter*, especially the *Acinetobacter lwoffii* species, was significantly enriched in OC tissues compared to normal distal fallopian tube tissues [21].

To explore the underlying mechanisms between *Acinetobacter* and epithelial OC, the following two aspects should be considered: bacterial factors and host responses against them. Bacterial products, such as lipopolysaccharide (LPS), can stimulate the tumor to produce proinflammatory cytokines that enhance tumor survival (LPS-induced tumor growth). On the host side, Toll-like receptors (TLRs) are transmembrane proteins known to play an important role in immunosurveillance and responses toward microorganisms [29].

Previously, through in vitro and in vivo studies, researchers have demonstrated that LPS, as well as EVs, secreted by *A. baumannii* stimulate the TLR-4 signaling pathway and trigger the host’s immune response against an *A. baumannii* infection [30,31,32,33]. In addition, EVs secreted by *Acinetobacter nosocomialis*, another important pathogen of various opportunistic infections, are also known to induce cytotoxicity of epithelial cells and host inflammatory responses [34]. Interestingly, the expression of TLR-4 is observed in both the normal ovarian surface and epithelial ovarian tumor cell lines [35]. In epithelial OC, TLR-4 signaling has been demonstrated to promote tumor growth and to develop chemoresistance [36]. Therefore, we suggest that products secreted by the *Acinetobacter* species may cause the development of epithelial OC through the TLR-4 signaling pathway.

In accordance with the era of precision medicine, it is obvious that reliable diagnostic tools are essential for detecting OC. Our study results imply that adding the metagenomic data to the conventional diagnostic model might improve its performance in the detection of OC. However, the diagnostic model composed of patients’ ages, serum CA-125 levels, and relative abundance of the genus *Acinetobacter* needs to be externally validated. Nevertheless, this study tried to overcome this limitation by separating the test and training sets from the beginning and faithfully implementing the internal validation.

Developing the diagnostic models for identifying OC, we believed that it was the most important to reduce the false-negative rate considering its worse prognosis compared to any other malignancies. Therefore, during the model construction, we focused on achieving a high accuracy and maintaining the sensitivity, even if specificity was compromised. As the result, we reported our newly developed diagnostic model’s diagnostic performance as follows: sensitivity 86.4% and specificity 78.4% (AUC 0.898) in the training set; and sensitivity 82.1% and specificity 68.0% (AUC 0.846) in the test set.

Diagnostic performance of our newly developed, microbiome biomarker-based diagnostic model was not compared with the currently available tools, such as the ROMA and RMI scoring systems. At our institution, the serum HE4 test is not routinely performed in women with adnexal masses. In our study population, only 53.3% (129/242) underwent the serum HE4 test, so that ROMA could be calculated. Owing to the retrospective study design, we were not able to retrieve all the preoperative transvaginal ultrasonography images, so that RMI scoring system could not be applied. Moreover, if microbiome biomarkers are integrated with ROMA or RMI, there is the possibility that the diagnostic performance for identifying OC might be much improved. Now, we are planning a prospective cohort study to validate the clinical usefulness of the serum-based metagenomic analysis in the diagnosis of OC. In that study, every single subject will undergo both ROMA and RMI for further investigation.

The current study also has other limitations. First, the relationship between the microbiome and OC should be further investigated. We do not know whether our findings could explain the pathogenesis of OC or were just a phenomenon in this cohort. The cause-and-effect relationship between differing microbiome compositions and OC should be investigated. Additional translational studies, such as hypothesis-proving cell-line or animal studies, are warranted. Second, the current study is a single-institution study requiring external validation in different study populations. For example, the proportion of clear cell carcinoma in the OC group was relatively high: 16.4% and 19.6% in the training and test sets, respectively. In this study, all patients were Korean, and according to the literature on histologic types of epithelial OC, ovarian clear cell carcinoma is more common in the East Asian population than in the Western population [37,38]. Therefore, OC groups from other regions or ethnicities with different proportions of histologic types might have different metagenomic profiles of serum EVs. Third, the the FIGO stage of the OC cases was not considered in developing the diagnostic models. Approximately 30% of OC patients in our study population had FIGO stage I disease. The extent of disease might affect the composition of the serum microbe-derived EVs. Therefore, it is necessary to compare OC patients’ metagenomic profiles by stages in a large-sized cohort. Lastly, the sample size for the benign ovarian tumor group was small, which resulted in quite different histologic types between the training and test sets, although we randomly divided the samples.

Despite these limitations, the current study was the first to characterize the metagenomic profiles of the serum microbial EVs in OC. Through evaluation of the serum microbiomes, we were able to build a diagnostic model for OC. The metagenomic analysis of serum microbiomes has several advantages, particularly the ease of sample collection, which suggests an increase of its usability.

## 4. Materials and Methods

This retrospective case-control study using metagenomics was conducted after obtaining approval from the Institutional Review Board of Seoul National University Hospital, Seoul, Korea (SNUH; No. 1612-102-816).

### 4.1. Study Population

Since June 2012, we have been collecting biological samples of patients scheduled to undergo surgery for adnexal masses for research purposes; under the patients’ written informed consent, blood samples and cancer tissues are obtained the day before surgery and at the time of surgery, respectively, and then stored at the Human Biobank of SNUH.

For the present study, we identified relevant patients and obtained their frozen serum samples from the Human Biobank. Inclusion criteria for the study population were as follows: (1) older than 18 years; (2) underwent surgery for an adnexal mass between June 2012 and February 2018; and (3) pathologically diagnosed with either epithelial OC or benign ovarian tumor. Patients with the following conditions were excluded: (1) diagnosed with any malignancy other than OC synchronously or before the surgery; (2) neoadjuvant chemotherapy or targeted therapy before surgery; (3) borderline ovarian tumors; and (4) severe comorbidities, such as end-stage renal disease, uncontrolled diabetes mellitus, or long-term corticosteroid use.

In total, 166 patients with OC and 76 patients with benign ovarian tumors were included in this study. Through review of the medical records, we collected baseline characteristics including the age at diagnosis, BMI, comorbidities, and initial serum CA-125 levels. We also reviewed all patients’ pathology results and collected information on the FIGO stage for the study group. Then, the patients’ clinicopathologic characteristics were compared between the OC group and the benign ovarian tumor group. Metagenomic profiling was carried out with the patients’ frozen serum samples according to the procedures described below.

### 4.2. EV Isolation and DNA Extraction from Serum Samples

We isolated EVs from the serum samples using the differential centrifugation method, as described previously [39]. In brief, serum samples were centrifuged at 3000 rpm for 15 min at 4 °C, and 100 uL of the supernatant was mixed with 1 × PBS, pH 7.4 (ML 008-01, Welgene, Republic of Korea). The floating particles were sunk through centrifugation at 10,000× *g* for 1 min at 4 °C. After centrifugation, bacteria and foreign particles were thoroughly eliminated by sterilizing the supernatant through a 0.22-um filter.

To extract the DNA from the EVs’ membranes, EVs separated from serum in the previous steps were boiled for 40 min at 100 °C. To eliminate the remaining floating particles and debris, the supernatant was collected after 13,000 rpm of centrifugation for 30 min at 4 °C. EVs’ DNA was extracted using a DNA isolation kit according to the standard protocol (PowerSoil DNA Isolation Kit, MO BIO, Carlsbad, CA, USA). The DNA from EVs in each sample was quantified by using the QIAxpert system (QIAGEN, Hilden, Germany).

### 4.3. Microbial Metagenomic Analysis

For 16S rDNA gene-based metagenomic analysis, bacterial genomic DNA was amplified with 16S_V3_f (5′-TCGTCGGCAGCGTCAGATGTGTATAAGAGACAGCCTACGGGNGGCWGCAG-3′) and 16S_V4_r (5′-GTCTCGTGGGCTCGGAGATGTGTATAAGAGACAGGACTACHVGGGTATCTAATCC-3′) primers, which are specific for the V3-V4 hypervariable regions of the 16S rDNA gene. The libraries were prepared using PCR products according to the MiSeq System guide (Illumina, San Diego, CA, USA) and quantified using a QIAxpert (QIAGEN, Hilden, Germany). Each amplicon was then quantified, and the equimolar ratio was set, pooled, and sequenced on a MiSeq (Illumina, San Diego, CA, USA) according to the manufacturer’s recommendations.

### 4.4. Analysis of Microbial Composition in the Microbiota

Paired-end reads that matched the adapter sequences were trimmed by Cutadapt (version 1.1.6) [40]. The resulting FASTQ files containing paired-end reads were merged with CASPER and then quality filtered with Phred (Q) score-based criteria described by Bokulich [41,42]. Any reads shorter than 300 bp after merging were also removed. To identify the chimeric sequences, a reference-based chimera detection step was conducted with VSEARCH against the Greengenes database [43]. Next, the sequence reads were clustered into operational taxonomic units (OTUs) using CD-HIT with a de novo clustering algorithm under a threshold of 97% sequence similarity. The representative sequences of the OTUs were finally classified using the Greengenes database (version 13.8) with UCLUST (parallel_assign_taxonomy_uclust.py script on QIIME (version 1.9.1) under default parameters) [44]. The Chao indices, an estimator of the richness of taxa per individual, were estimated to measure the diversity of each sample.

### 4.5. Development of Diagnostic Models for Ovarian Cancer

To construct and validate the diagnostic models for OC, we randomly divided the samples from each group into training and test sets in the ratio 2:1, considering the ratio of OC and benign ovarian tumors in the total 242 samples. The values of each training and test set were transformed to a centered log ratio. Discovery of microbiome biomarkers and construction of diagnostic models were performed in the training set (*n* = 161), while validation of newly developed diagnostic models were performed in the test set (*n* = 81).

We filtered the genus if the zero proportion was more than 99%. To identify specific microbiome biomarkers that were differentially distributed between the OC and benign ovarian tumor groups, we performed metagenomic analyses using eight statistical methods popularly used with the filtered count data: Wilcoxon, Metastats, EdgeR, DESeq2, ZIG, ZIBSeq, ANCOM, and CLR Perm. We used the abundance of the OTUs as the algorithms were developed based on the abundance data. Comparing the list of significant microbiome biomarkers identified by each statistical method, we chose biomarkers that overlapped as far as possible, because each method provides a different list of microbiome biomarkers, and most overlapped ones are expected to be highly plausible biomarkers.

We constructed several diagnostic models identifying OC from benign ovarian tumors by combining the microbiome biomarkers with patients’ ages and serum CA-125 levels, and these models were validated in the test set. To evaluate the diagnostic performance of the developed models, each model’s sensitivity, specificity, and AUC were calculated.

### 4.6. Statistical Analysis

Statistical analyses were performed to evaluate differences in the clinicopathologic characteristics between the two groups. The Student’s *t*-test and Mann–Whitney U test were used to compare continuous variables, while the Pearson’s chi-square test and Fisher’s exact test were used to compare categorical variables. Shannon index was calculated to measure α-diversities of the microbiota.

Summaries of the eight statistical methods that were applied to the metagenomic analyses are as follows: (1) The Wilcoxon rank sum test is the nonparametric type of the two-sample *t*-test, which uses the sum of ranks for observations. (2) Metastats compares the number of samples by group and the number of taxaons. Welch’s *t*-test statistics were applied when the taxon count was larger than the number of samples. Otherwise, Fisher’s exact test was used. (3) EdgeR and (4) DESeq2 methods are usually used in the analysis of RNA-sequencing data. As metagenome data is extracted from 16S rDNA, application of these methods has been often tried. Both methods are the negative binomial models; however, the difference between the two methods is that EdgeR uses a trimmed mean of M-values normalization, whereas DESeq2 uses a relative log expression normalization. (5) ZIG uses the log normal mixture model for the taxon count, taking sparsity on the OTU table into account. To overcome high false-positive rates, we adopted empirical Bayes shrinkage of parameter estimates. (6) ZIBSeq uses the beta mixture model for relative abundance. Relative abundance after total sum-scaling normalization was performed owing to the large number of zeros and results with the skewed distribution. (7) ANCOM was used to compare relative abundance of the OTUs; Wilcoxon rank sum test was used in comparisons of the two groups after the log-ratio transformation of all pairwise taxa. The Kruskal-Wallis test was used in comparisons of the three groups, and the Freidman test was used in comparisons of repetitive data. (8) CLR Perm fits the logistic model after the centered log-ratio transformation with count data to alleviate the sum to one constraint of relative abundance. The permutation test was adopted to decrease the false discovery rate.

R statistical software (version 3.4.4; R Foundation for Statistical Computing, Vienna, Austria; ISBN 3-900051-07-0; http://www.R-project.org) was used for the statistical analyses. A two-sided *p*-value below 0.05 was considered statistically significant.

## 5. Conclusions

In conclusion, we found that 16S rDNA gene-based metagenomic analyses revealed differences in the metagenomic profiles of serum microbial EVs between patients with OC and those with benign ovarian tumors. We also developed a microbiome biomarker-based diagnostic model differentiating OC from benign ovarian tumors and found that the serum microbiome may play a role in the early detection of OC. Further prospective studies are warranted to validate these results.

## Figures and Tables

**Table 1:** Patients’ clinicopathologic characteristics.

Characteristics	All (*n* = 242, %)	Training Set			Test Set		
		Cancer (*n* = 110, %)	Benign (*n* = 51, %)	*p*	Cancer (*n* = 56, %)	Benign (*n* = 25, %)	*p*
**Age, years**							
Mean ± SD	52.3 ± 13.4	53.8 ± 12.3	48.2 ± 15.9	0.031	53.4 ± 11.5	51.8 ± 15.7	0.658
BMI, kg/m2							
Mean ± SD	23.0 ± 3.4	22.6 ± 3.1	23.1 ± 3.6	0.387	23.1 ± 3.6	23.9 ± 4.2	0.370
Menopause	141 (58.3)	68 (61.8)	26 (51.0)	0.194	34 (60.7)	13 (52.0)	0.463
Comorbidities							
Hypertension	55 (22.7)	27 (24.5)	8 (15.7)	0.205	11 (19.6)	9 (36.0)	0.115
Diabetes	21 (8.7)	11 (10.0)	6 (11.8)	0.735	1 (1.8)	3 (12.0)	0.085
Dyslipidemia	34 (14.0)	18 (16.4)	6 (11.8)	0.446	6 (10.7)	4 (16.0)	0.489
Serum CA-125, IU/mL							
Median (range)	126.3 (2.3–10000.0)	331.1 (2.3–10000.0)	22.3 (3.5–1821.0)	<0.001	432.3 (7.7–9909.0)	20.6 (5.7–1710.0)	<0.001
FIGO stage							
I	52 (21.5)	33 (30.0)			19 (33.9)		
II	10 (4.1)	6 (5.5)			4 (7.1)		
III	75 (31.0)	53 (48.2)			22 (39.3)		
IV	29 (12.0)	18 (16.4)			11 (19.6)		
Histologic type							
***Epithelial ovarian cancer***							
High-grade serous	88 (36.4)	60 (54.5)			28 (50.0)		
Low-grade serous	8 (3.3)	6 (5.5)			2 (3.6)		
Mucinous	15 (6.2)	10 (9.1)			5 (8.9)		
Endometrioid	16 (6.6)	9 (8.2)			7 (12.5)		
Clear cell	29 (12.0)	18 (16.4)			11 (19.6)		
Mixed	6 (2.5)	3 (2.7)			3 (5.4)		
Others	4 (1.7)	4 (3.6)			0		
***Benign ovarian tumor***							
Mucinous cystadenoma	28 (11.6)		24 (47.1)			4 (16.0)	
With fibroma	5 (2.1)		4 (7.8)			1 (4.0)	
Without fibroma	23 (9.5)		20 (39.2)			3 (12.0)	
Serous cystadenoma	15 (6.2)		8 (15.7)			7 (28.0)	
With fibroma	4 (1.7)		3 (5.9)			1 (4.0)	
Without fibroma	11 (4.5)		5 (9.8)			6 (24.0)	
Seromucinous cystadenoma	6 (2.5)		4 (7.8)			2 (8.0)	
With fibroma	2 (0.8)		1 (2.0)			1 (4.0)	
Without fibroma	4 (1.7)		3 (5.9)			1 (4.0)	
Endometriotic cyst	8 (3.3)		4 (7.8)			4 (16.0)	
Mature cystic teratoma	8 (3.3)		6 (11.8)			2 (8.0)	
Fibroma/fibrothecoma	9 (3.7)		3 (5.9)			6 (24.0)	
Paratubal cyst	2 (0.8)		2 (3.9)			0	

Abbreviations: BMI, body mass index; CA-125, cancer antigen 125; FIGO, International Federation of Gynecology and Obstetrics; and SD, standard deviation.

**Table 2:** Top 10 genus-level microbiome biomarkers significantly differentially distributed between the ovarian cancer and benign ovarian tumor groups.

Genus	Wilcoxon	Metastats	EdgeR	DESeq2	ZIG	ZIBSeq	ANCOM	CLR Perm
*Acinetobacter*	<0.001	0.008	0.093	0.043	<0.001	<0.001	*Acinetobacter*	<0.001
*Isoptericola*	0.841	<0.001	0.487	1	0.046	<0.001	Not detected	0.855
*Terrisporobacter*	0.841	0.023	0.600	1	<0.001	<0.001	Not detected	0.944
*SM1A02*	0.989	0.008	0.528	1	<0.001	0.002	Not detected	0.935
*Candidatus Alysiosphaera*	0.841	<0.001	0.476	1	0.015	<0.001	Not detected	0.901
*Ralstonia*	0.841	<0.001	0.462	1	<0.001	0.005	Not detected	0.913
*Hydrogenophaga*	0.771	<0.001	0.872	1	<0.001	0.027	Not detected	0.809
*Pseudorhodoferax*	0.921	0.024	0.811	1	<0.001	0.007	Not detected	0.779
*Bryobacter*	0.841	0.023	0.420	1	0.007	0.999	Not detected	0.849
*Varibaculum*	0.841	0.013	0.600	1	0.599	<0.001	Not detected	0.416

Shown with the *q* values.

**Table 3:** Diagnostic models differentiating ovarian cancer from benign ovarian tumors.

Model	Training Set			Test Set		
	Sensitivity	Specificity	AUC	Sensitivity	Specificity	AUC
Age	0.554	0.490	0.589	0.518	0.520	0.531
Age, CA-125	0.773	0.686	0.809	0.768	0.560	0.816
Age, *Acinetobacter*	0.827	0.529	0.770	0.839	0.440	0.667
Age, CA-125, *Acinetobacter*	0.864	0.784	0.898	0.821	0.680	0.846

Abbreviations: AUC, area under the receiver operating characteristic curve (AUC) and CA-125, cancer antigen 125.

## Supplementary Materials

The following are available online at https://www.mdpi.com/2072-6694/12/5/1309/s1: Figure S1. Flow diagram illustrating the overall study design. Figure S2. Comparisons of genus-level α-diversity between the two groups. Figure S3. Genus-level multidimensional plots.
