# Supplementary material for: Metagenomic Analysis of Serum Microbe-Derived Extracellular Vesicles and Diagnostic Models to Differentiate Ovarian Cancer and Benign Ovarian Tumor
Source: Cancers (Basel). 2020 May 21;12(5):1309. doi: 10.3390/cancers12051309 (PMC7281409; doi:10.3390/cancers12051309)
Supplement: Supplementary file 1 [file cancers-12-01309-s001.pdf]

Supplementary Materials: Metagenomic Analysis of Serum Microbe-Derived Extracellular Vesicles and Diagnostic Models to Differentiate Ovarian Cancer and Benign Ovarian Tumor

Se Ik Kim, Nayeon Kang, Sangseob Leem, Jinho Yang, HyunA Jo, Maria Lee, Hee Seung Kim, Danny N. Dhanasekaran, Yoon-Keun Kim, Taesung Park and Yong Sang Song

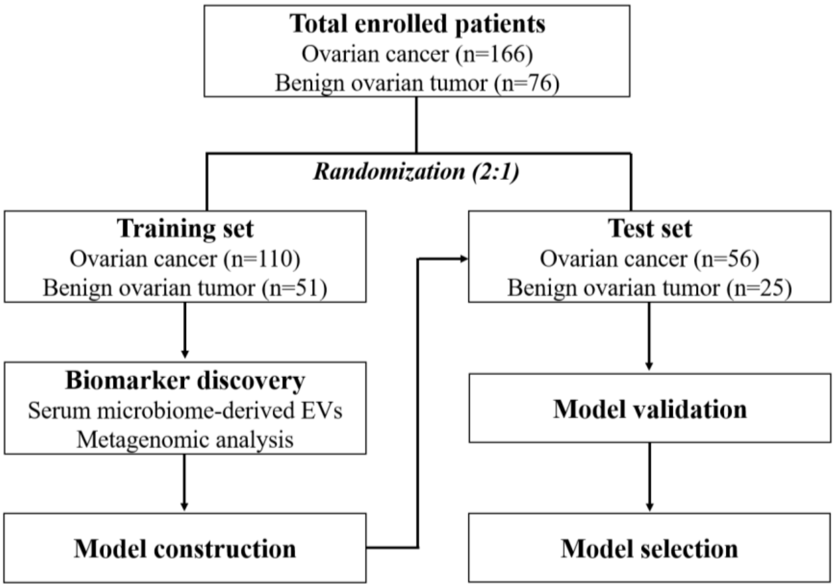

Figure S1. Flow diagram illustrating the overall study design.

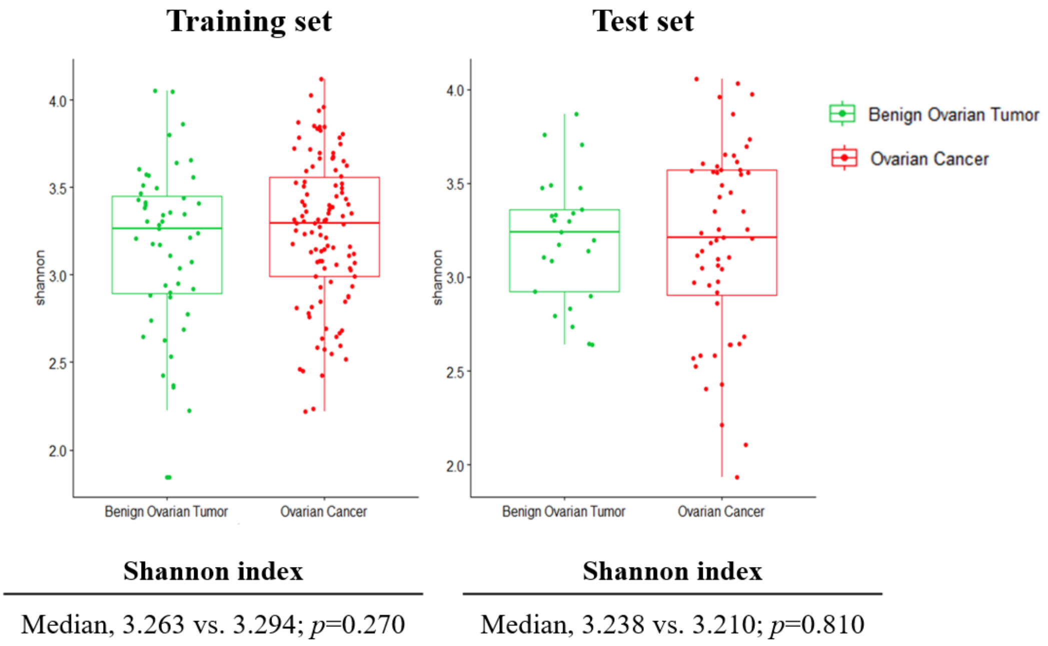

Figure S2. Comparisons of genus-level α-diversity between the two groups. (left)Training set. (right) Test set.

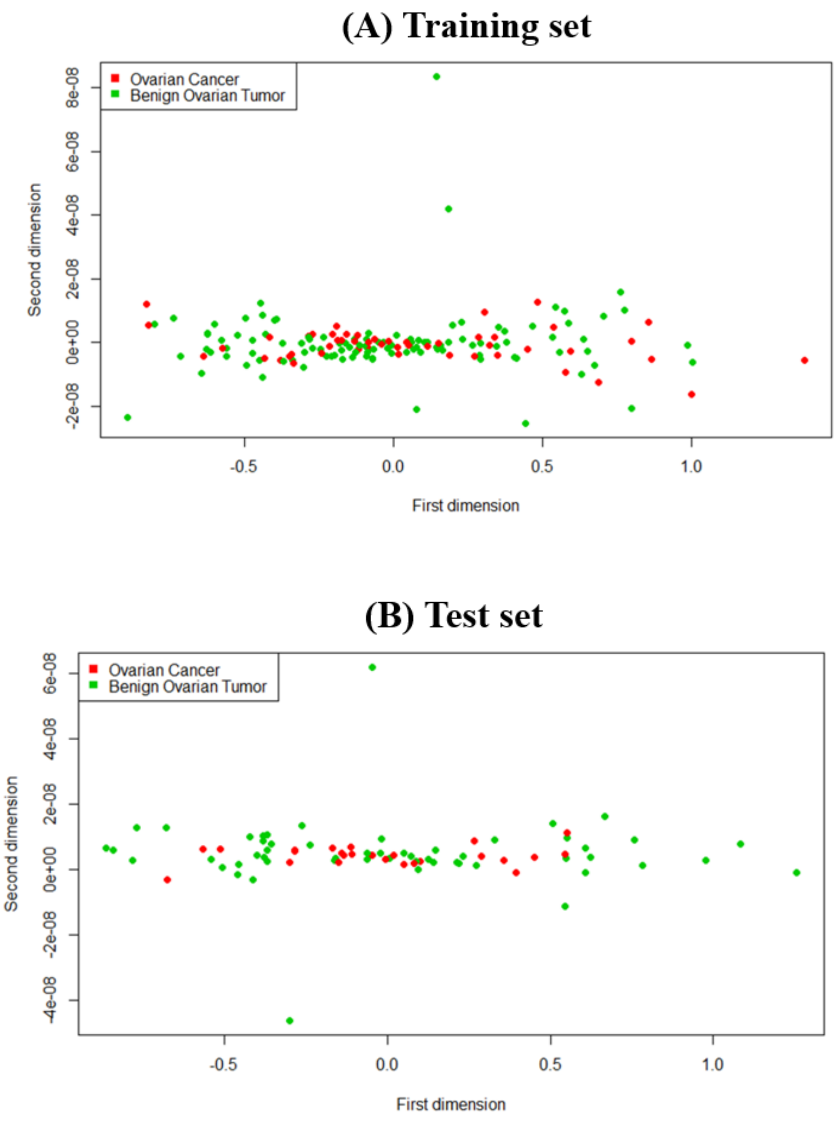

Figure S3. Genus-level multidimensional plots. (**A**) Training set. (**B**) Test set.

© 2020 by the authors. Licensee MDPI, Basel, Switzerland. This article is an open access article distributed under the terms and conditions of the Creative Commons Attribution (CC BY) license (http://creativecommons.org/licenses/by/4.0/).
